# Effects of Prenatal Exposure to Alcohol Across the Life Span

**Published:** 1996

**Authors:** Paul D. Connor, Ann P. Streissguth

**Affiliations:** Paul D. Connor, Ph.D., is a senior fellow and Ann P. Streissguth, Ph.D., is a professor at the Fetal Alcohol and Drug Unit, Department of Psychiatry and Behavioral Sciences, University of Washington, Seattle, Washington

**Keywords:** prenatal alcohol exposure, fetal alcohol effects, fetal alcohol syndrome, diagnostic criteria, congenital anomaly, neurodevelopmental anomaly, cognitive process, brain damage, attention, memory, intelligence and ability, motor coordination, problem solving, treatment

## Abstract

Prenatal exposure to alcohol can have many detrimental effects throughout the life span. Of primary concern are changes in the brain that lead to deficiencies in cognitive functioning, including memory and learning problems, attention deficits, poor motor coordination, and difficulties with problem-solving. These cognitive deficiencies create long-standing problems in many spheres of life, including disturbances in work, school, and social functioning. Treatment strategies that have been used with other cognitively impaired populations may be adapted to assist patients who display the various cognitive symptoms associated with prenatal alcohol exposure.

Maternal alcohol consumption can affect the development of both the body and the brain of the fetus, with consequences that may persist throughout life. Only recently have researchers begun to evaluate the effects of prenatal alcohol exposure on performance in school and the workplace as the patient passes through childhood and adulthood. This article describes some types of structural brain damage that occur in humans and animals prenatally exposed to alcohol, reviews ways in which cognitive functioning can be impaired by fetal alcohol exposure, and discusses prevention and treatment strategies.

This article focuses on readily diagnosable syndromes of brain dysfunction brought about by high levels of prenatal alcohol exposure. For studies that focus on the more subtle effects of lower levels of exposure, see Coles (1992), Day and Richardson (1991), and Streissguth and colleagues (1993).

## Nomenclature and Diagnostic Criteria

Many labels have been used to describe the effects of significant intrauterine alcohol exposure. Fetal alcohol syndrome (FAS) (Jones and Smith 1973) is a medical diagnosis initially defined as a specific pattern of facial and other physical deformities occurring in conjunction with growth retardation. Sokol and Clarren (1989) refined this definition and suggested that to support the diagnosis, effects must be observable in all three of the following realms: overall growth retardation, structural or functional abnormalities of the brain, and a characteristic pattern of facial deformities. The brain abnormalities may include structural and cognitive defects, delayed brain development, and signs of neurological impairment. The facial features characteristic of the young child with FAS are depicted in the figure.

The diagnosis of FAS identifies only a relatively small proportion of children prenatally affected by alcohol. Therefore, the terms “fetal alcohol effects” (FAE) and “possible fetal alcohol effects” (PFAE) have been used since the mid-1970’s to describe patterns of birth defects following significant prenatal alcohol exposure that do not include all of the facial features or growth retardation seen in FAS (Clarren and Smith 1978). In addition, Sokol and Clarren (1989) used the category “alcohol-related birth defects” (ARBD) to focus primarily on the physical anomalies rather than the brain disturbances associated with severe prenatal alcohol exposure.

A recent report from the Institute of Medicine (IOM) suggests a new category of prenatal alcohol exposure that would essentially replace FAE (IOM 1996). The classification, referred to as “alcohol-related neurodevelopmental disorder” (ARND), focuses specifically on brain dysfunctions in the presence of significant prenatal alcohol exposure. IOM suggests using this term for patients who have a documented prenatal exposure to alcohol and identifiable problems suggesting faulty brain development. Unlike FAS, ARND does not require the presence of facial or other physical anomalies. Other diagnostic schemes are also being developed. Whenever possible, this article uses the diagnosis of FAS as defined by Sokol and Clarren (1989) and the diagnosis of ARND as defined by IOM (1996). However, the term “FAE” is retained when it is the classification used by a cited reference.

The range of prenatal alcohol effects has been described as a continuum, with no effects at one end and full developed FAS at the other end. This view, however, can be misleading. Although patients with FAS have more physical deformities, such as heart malformations and facial anomalies, the brain dysfunctions of people with FAE/ARND are often as severe as—if not worse than—brain dysfunctions in patients with FAS.

**Figure:**
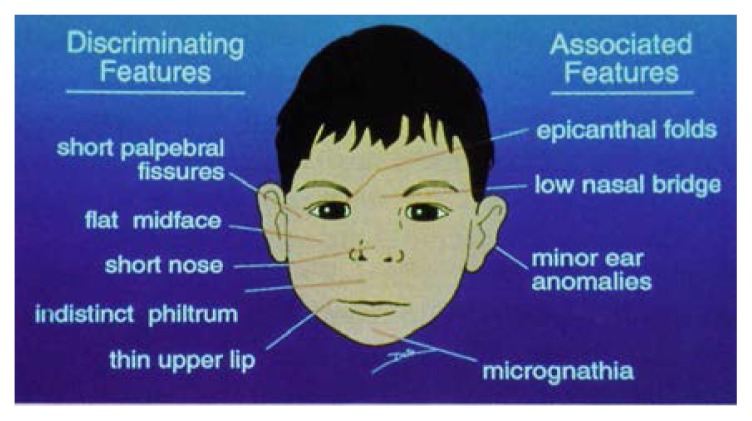
Facial features particularly characteristic of the FAS child. Discriminating features (i.e., those considered definitive signs of FAS) are shown on the left side of the illustration; characteristics listed on the right side are associated with FAS but are not sufficient to determine the presence of the syndrome. Microcephaly (small head circumference) is not a facial feature per se, but a central nervous system characteristic. (Epicanthal folds = skin folds covering inner corner of the eye; philtrum = groove between nose and upper lip; micrognathia = abnormal smallness of the jaws; palpebral fissures = eye openings.) SOURCE: Streissguth and Little 1994.

Although no alcohol-dose threshold is known to differentiate between FAS and ARND, the time of alcohol ingestion appears to be important. Drinking relatively early in pregnancy may lead to many of the facial anomalies seen in children with FAS, whereas the deleterious effects of alcohol on the brain can occur at any time during pregnancy.

## Structural Damage to the Brain

In seeking to understand the brain dysfunctions that occur in patients diagnosed with FAS or ARND, researchers have studied brain structures in both animal models and human patients. The structural changes observed through this research can be related to defects in brain function.

### Animal Studies

Animal models of FAS and ARND demonstrate widespread damage to the brain following relatively high prenatal exposure to alcohol, as well as significant brain changes following even moderate alcohol exposure. These changes in brain structure also have been identified frequently in human patients with FAS/FAE. In general, alcohol-exposed rats have smaller and lighter brains (Goodlett and West 1992). Specific brain structures affected by prenatal exposure include the basal ganglia and the cerebellum, which are small in cases of exposure. Increases in the size of the fluid-filled cavities in the brain (i.e., the ventricles) also have been observed (Goodlett and West 1992; Mattson et al. 1994; Bonthius et al. 1996). Other studies have reported an overall reduction in the number of cells in the cerebral cortex, damage to a particular type of cell (i.e., pyramidal cells) in the hippocampus, and damage to the main pathway for the sense of smell in rats (Riley et al. 1986; Goodlett and West 1992).

### Human Studies

Many of the structural changes detected through animal studies also have been found in studies on humans. For example, children diagnosed with FAS often have a smaller-than-expected brain size and a smaller head (Mattson and Riley 1995), as well as reduction in the size of the cerebellum and the structures involved in the sense of smell. Ventricles may be enlarged, small, or absent (Mattson and Riley 1996).

The basal ganglia also have been identified as a site of cellular damage and shrinkage in humans with FAS (Mattson and Riley 1995). The communication pathways interconnecting the brain’s two hemispheres (i.e., the corpus callosum) have been found to be atrophied or absent in many of these patients. Microscopic changes have been reported in the location of cells within the brain, suggesting evidence of abnormal migration patterns. As the fetus develops, cells move from one part of the brain to another in a prespecified pattern. If this migration is in some way disrupted, as it can be with prenatal alcohol exposure, the cells may not reach their intended destination; as a result, these cells may not function properly (see Michaelis and Michaelis 1994).

Although animal studies have shown prenatal alcohol-related changes in some regions of the brain involved with memory functioning (e.g., the hippocampus), little analogous literature exists for humans, perhaps because it has been difficult to measure these structures reliably. However, new imaging techniques may reveal alcohol-related damage to these brain structures as well (Hopkins et al. 1996).

## Neuropsychological Deficits

Scientists have recently begun investigating neuropsychological deficits in subjects diagnosed with FAS/ARND. Most of these studies have focused on children and adolescents, although researchers are increasingly reporting the persistence of deficits into adulthood. Because prenatal alcohol exposure affects so many regions of the brain, patients exhibit a wide variety of disturbances. Problems occur in a number of important areas, including attention, intelligence, memory, motor coordination, complex problem-solving, and abstract thinking.

Studies of infants reveal tremors, difficulty “tuning out” redundant sensory stimuli, and a weak suckle. Infants and toddlers may be developmentally delayed and often are hyperactive. As the child grows, various tests can help researchers investigate cognitive and behavioral problems. These tests, which can take from 1 hour to 1 full day to conduct, may be developed from a standard battery of neuropsychological tests or tailored to the specific neuropsychological problems being studied.

Neuropsychological tests show that people with FAS and ARND often have a hard time focusing their attention. Nanson and Hiscock (1990) found that 5- to 12-year-old children with FAS/FAE exhibited attentional deficits similar to children diagnosed with attention deficit disorder. Attentional disturbances also were prominent in adolescents diagnosed with FAS (Carmichael Olson et al. 1992*b*). These adolescents made impulsive errors on vigilance tasks, which required focusing and sustaining attention. In a study of caretaker reports about subjects with FAS/FAE (Streissguth et al. 1996), 60 percent of the subjects who were 6 to 11 years old and 60 percent of those who were 12 to 20 years old were reported to have had attention deficit problems in their lives. Caretakers in this study were defined as biological, adoptive, or foster parents; other people who were closely involved in the care of the subject; or those who reported a strong familiarity with the subject’s history. For the adults with FAS or FAE, attentional problems were reported at some time in their lives by more than 40 percent of the caretakers. (This figure may be misleadingly low, because some informants had not known the subjects as children.) Patients who have difficulty focusing and maintaining attention may function poorly in work and in school. They may become distracted by extraneous events, losing track of the task at hand. For example, 70 percent of the subjects studied by Streissguth and colleagues (1996) had recurring problems paying attention at school.

Intellectual functioning, as measured by standardized IQ tests, is often below average in children and adolescents with FAS or FAE (Conry 1990). Streissguth and colleagues (1996) found that 68 percent of FAS/FAE subjects in the three age groups studied (i.e., children, adolescents, and adults) had received services for learning problems in school.

Performance in arithmetic is especially poor in patients with FAS/FAE (Kerns et al. in press). In a recent study by Kopera-Frye and colleagues (in press), adolescent and adult patients with FAS and FAE had significant difficulty performing tasks involving the calculation and estimation of numerical concepts, although they could read and write numbers. This deficit could make tasks that are important for independent living, such as managing finances, difficult. In a recent study, more than 80 percent of adult subjects with FAS/FAE were reported as sometimes or frequently needing help managing money (Streissguth et al. 1996).

Subjects with FAS/ARND often have problems with memory. Kerns and colleagues (in press) found that patients with FAS had difficulty recalling a list of words even after hearing the list five times. These patients often failed to cluster words into categories (i.e., tools or fruit), a strategy that facilitates recall. They also made more “intrusive” responses, adding words that were not on the original list. Mattson and colleagues (1994) hypothesized that patients with FAS performed on list-learning tasks similarly to patients with Huntington’s disease. (Huntington’s disease is a degenerative brain disorder that affects the basal ganglia, producing movement abnormalities and dementia.) Both FAS and Huntington’s subjects made errors consisting of repeating the same word over and over (i.e., perseverative errors). In addition, they functioned better when asked to recognize a list of previously presented words from a much longer list than when asked to recall the list without the benefit of reminders. The authors concluded that these characteristics may be explained by abnormalities in the basal ganglia.

The difficulties in memory functioning that have been observed suggest that people with FAS/FAE may often forget their obligations at school or work, possibly resulting in disruptions of education or termination of employment. They also may forget medical appointments and thus may not receive timely medical care. Because of this problem, people with FAS/FAE often require someone else to remind them of appointments. Uecker and Nadel (1996) found that children diagnosed with FAS experienced significant difficulty in remembering objects and produced many more spatial distortions when drawing than did control subjects. The researchers concluded that the difficulties in object memory were similar to the performance of patients who had undergone removal of the right temporal lobe of the brain, including the right hippocampal formation, to help control epilepsy. The authors therefore speculated that patients with FAS may have right hippocampal dysfunction.

Janzen and colleagues (in press) found that 3^1^/_2_- to 5-year-old children with FAS were significantly deficient in the ability to integrate visual and motor functions, although their visual perception abilities appeared to be normal. Other studies (e.g., Marcus 1987) have reported deficits in balance and fine motor coordination in FAS children, suggesting cerebellar dysfunction. Subjects with FAS/FAE may therefore appear clumsy, especially when attempting fine motor manipulations.

Problem-solving, concept formation, and verbal fluency have been subjects of recent study in children and adolescents with FAS or ARND. Studies have found that FAS children and adolescents have difficulty solving problems and shifting attention between tasks and tend to make perseverative responses. These subjects also complete fewer categories on a test that measures these abilities (i.e., the Wisconsin Card Sorting Test) than do normal children (Carmichael Olson et al. 1992*b*; Kodituwakku et al. 1995). Several studies have found that subjects with FAS/FAE have decreased verbal fluency (i.e., naming as many words as possible in a given time) and nonverbal fluency (i.e., drawing as many designs as possible in a given time). They also have difficulty with cognitive estimation, a category of tasks that require the subject to estimate sizes, weights, amounts, and lengths of items to which they may not know the exact answer, such as “What is the height of the tallest tree in the world?” or “What is the age of the world’s oldest woman?” People with FAS/FAE tend to give more extreme answers to many of these types of questions than may be expected. For example, they may report the length of a dollar bill as five feet (Kodituwakku et al. 1995; Kerns et al. in press; Kopera-Frye et al. in press). Deficits in these areas may make it difficult for the person with FAS/FAE to understand and learn complicated activities that require abstract reasoning to help them cope in complicated work and school environments.

## Treatment Strategies

Although there have been no systematic studies of the benefits of early intervention for infants and young children with FAS/FAE, some approaches may be helpful. First, it is imperative that interventions include the whole family. Parents of children with FAS/FAE must be helped to understand that the behavioral and cognitive problems that arise are not something that the child is consciously choosing to do. Intervention also includes assisting the family in gaining access to special education, vocational training, and other services (Streissguth in press). Hand-in-hand with early intervention is early diagnosis. Parents report that an early diagnosis has been helpful to them in setting appropriate expectations for their child’s performance.

So far, no empirical research provides insight on how to ameliorate the specific cognitive disturbances accompanying FAS and ARND. However, research addressing rehabilitation strategies for other neuropsychologically impaired populations may be relevant (Sohlberg and Mateer 1989). For example, cognitive rehabilitation approaches (i.e., the use of compensation strategies for areas of deficit and attempts to ameliorate the deficit directly) are frequently used for patients with traumatic brain injury and may benefit people with FAS/FAE. In addition, anecdotal reports suggest that behavioral strategies using high levels of structure, concrete (rather than abstract) rules and consequences, and close caretaker supervision may help the FAS patient (Carmichael Olson et al. 1992*a*).

Behavioral approaches that assist children with FAS/FAE in learning more adaptive ways of communicating their needs or feelings may help minimize the negative, immature, and attention-getting behaviors that are frequently observed in such children (Burgess and Streissguth 1992). One of these approaches, positive behavioral management, involves bringing about behavioral change through systematic management of the behavioral consequences. Although positive behavioral management strategies have been systematically applied to hundreds of autistic children, no analogous scientific literature exists on children with FAS/FAE. Dyer and colleagues (in press) describe one case of an 18-year-old with FAS and the behavioral techniques that were developed to change his behaviors.

Kleinfeld and Wescott (1993) suggest that integration and coordination of the family, schools, and other community services can be beneficial in working with the child. These authors include anecdotal reports from parents and teachers about strategies that have helped children with FAS. Other similar guides have been developed that suggest tactics that may be helpful when working with younger children (see, for example, Villarreal et al. 1992). Finally, medications are another avenue of intervention that needs to be systematically examined, particularly in terms of the high frequency of attentional problems in children with FAS/ARND and the significant levels of depression noted in adolescents and adults.

## Prevention of FAS/ARND

Prevention of future occurrences is a vital aspect of the study of FAS. Informational programs that increase awareness of the damaging effects of drinking during pregnancy allow women to make more informed choices. Informing primary care providers about the effects of maternal alcohol use facilitates the identification of women who may be at risk for alcohol abuse during pregnancy, helps these women get services for their alcohol problems, and aids in early identification of children who may have been prenatally exposed. Targeting high-risk mothers and helping them obtain appropriate services is important in preventing new cases of FAS/ARND (see, for example, Weiner and Larsson 1987 and Grant et al. 1996).

## Conclusions

Prenatal exposure to alcohol can have many deleterious effects throughout the life span. Patients with FAS show evidence of structural changes both facially as well as in the brain. Of primary concern are the changes in the brain that lead to deficits in cognitive functioning, including memory and learning problems, attention problems, coordination problems, and difficulties with problem-solving. Unlike the facial features, which tend to become more normal as the affected child grows to adulthood, the cognitive deficits persist, creating long-standing problems in many spheres of life. Affected individuals experience difficulties in work, school, and social functioning. Further study is needed to address the persistence of cognitive and emotional problems throughout the life span of people with FAS, particularly for adults with FAS/ARND, because many of the infants that were first diagnosed in the early 1970’s are now adults. Finally, systematically examining various treatment and intervention strategies that have been used for other populations is vitally important in helping to tailor home, school, and community treatment programs to the problems that are commonly found in people with FAS and ARND.
